# The Use of Liquids Ionic Fluids as Pharmaceutically Active Substances Helpful in Combating Nosocomial Infections Induced by *Klebsiella Pneumoniae* New Delhi Strain, *Acinetobacter Baumannii* and *Enterococcus* Species

**DOI:** 10.3390/ijms19092779

**Published:** 2018-09-15

**Authors:** Andrzej Miskiewicz, Piotr Ceranowicz, Mateusz Szymczak, Krzysztof Bartuś, Paweł Kowalczyk

**Affiliations:** 1 Department of Periodontology and Oral Diseases, Medical University of Warsaw, 18 Miodowa St., 00-246 Warsaw, Poland; andrzej.miskiewicz@wum.edu.pl; 2 Department of Physiology, Faculty of Medicine, Jagiellonian University Medical College, 31-531, Cracow, Poland; piotr.ceranowicz@uj.edu.pl; 3 Department of Microbiology, Institute of Agricultural and Food Biotechnology, 36 Rakowiecka St., 02-532 Warsaw, Poland; m.szymczak@ibprs.pl; 4 Department of Applied Microbiology, Faculty of Biology, Institute of Microbiology, University of Warsaw, Miecznikowa 1 St., 02-096 Warsaw, Poland; 5 Department of Cardiovascular Surgery and Transplantology, Faculty of Medicine, Jagiellonian University, JP II Hospital, 80 Prądnicka St., 31-202 Krakow, Poland; krzysztof.bartus@uj.edu.pl; 6 Department of Animal Nutrition, The Kielanowski Institute of Animal Physiology and Nutrition, Polish Academy of Sciences, 05-110 Jabłonna, Poland

**Keywords:** Ionic liquids, drug resistance, antimicrobial effect

## Abstract

This review deals with various microbiological activities of ionic liquids, which constitute the first anti-infective defense against multi-drug-resistant bacteria—with a particular emphasis placed on medicine and pharmacology. The quoted data on the biological activity of ionic liquids including their antimicrobial properties (depending on the type of a cation or an anion) and are discussed in view of possible applications in nosocomial infections. Dedicated attention is given to finding infections with the *Klebsiella pneumoniae* New Delhi strain, *Acinetobacter baumannii*, and *Enterococcus* species, which are responsible for the induction of antibiotic resistance in intensive care units. Diagnosis and treatment using current antibiotics is a significant problem in hospital care, and the relevant burden on the health systems of the European Union member states induces the search for new, effective methods of treatment. Ionic liquids, due to their antibacterial effect, can be considered topical and general medications and may provide the basis for treatment to eliminate the antibiotic resistance phenomenon in the future. At present, the number of infections with resistant pathogens in hospitals and outpatient clinics in the European Union is growing. In 2015–2017, a significant incidence of respiratory and bloodstream infections with bacteria resistant to antibiotics from the 3rd generation group of cephalosporins, glycopeptides, and carbapenems were observed. The paper presents examples of synthesized bifunctional salts with at least one pharmaceutically active ion in obtaining a controlled release, controlled delivery, and biological impact on the pathogenic bacteria, viruses and fungi. The ionic liquids obtained in the presented way may find applications in the treatment of wounds and infections.

## 1. Introduction 

**Figure 1:**
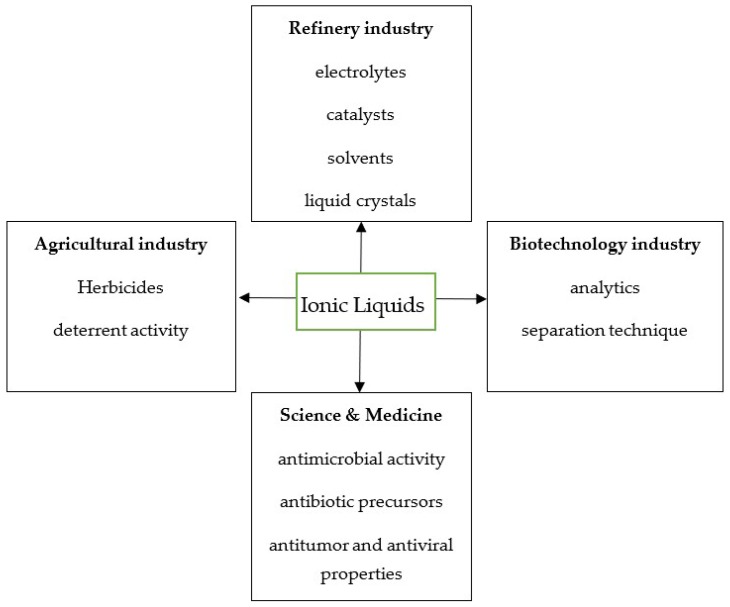
The chart summarizes main properties and their current applications of ionic liquids [8,9,10,11].

The name ionic liquids (ILs) appeared for the first time in the 1970s, and in the literature, the term began to be used only in the mid-1990’s [1]. Previously, the term “molten salts” was used. ILs form metastable structures (supercooled liquids and vitreous states) and melt below 100 °C—the boiling point of water. Most salts, however, melt at high temperatures (e.g., sodium chloride at 800 °C) [2]. There are also salts that melt at temperatures below room temperature (below 20 °C), which are referred to as RTIL (room temperature ionic liquids) [3]. ILs are defined as liquid chemical substances consisting solely of ions: An organic cation and an organic or inorganic anion [4]. Examples of organic anions are acetates, lactates, salicylates, benzoates, saccharinates, and thiazolanes [5]. ILs with a thiazolate anion are classified as energetic ionic liquids. The anions of inorganic origin with simple structures include Cl^−^, Br^−^, I^−^, NO_2_^−^, NO_3_^−^, and SO_4_^−^ with a complex structure, and depending on the number of central atoms, one-core anions are included, such as BF_4_^−^, PF_6_^−^, ZnCl_3_^−^, CuCl_2_^−^, SnCl_3_^−^, and AlCl_4_^−^, as well as multicore anions like A_12_C_17_^−^, Al_3_Cl_10_^−^, and Fe_2_Cl_7_^−^ [6,7]. The universal ability to modify the chemical structure of ILs allows the selection of the right chemical compound that can be used as a targeted agent for bacterial infections and microbiology, medicine, pharmacy, and industry, which in combination with occurrence in the liquid state of aggregation over a wide range of temperatures, determines their technological usability (Figure 1).

### 1.1. Physico-Chemical Properties of ILs in Antimicrobial Activity

Ionic liquids attract the attention of the pharmaceutical industry due to their unique properties. Both thermodynamics and kinetics of the ionic liquids differ from conventional solvents [12]. Conducting a reaction in an ionic liquid is often easier and faster than in a conventional reaction environment, and besides, the application of green solvents does not require any special methodology [10]. The ILs may constitute a new alternative to antibiotics, therefore, attention should be paid to their specific physical and chemical properties like: Volatility [2], viscosity [13,14,15,16], nonflammability and negligible vapor pressure for measuring thermodynamic properties [17], melting temperature [12], solubility and stability at high temperatures, and surface activity [18]. An important characteristic of ILs is that the ionic liquid applied can be easily separated from the reactive environment by rinsing with water and subsequently by vaporizing the solvent under a vacuum [19]. An additional asset of ILs resulting from their thermodynamic properties is their ability to form multiphase systems, which has been used for the liquid-liquid erythromycin antibiotic extraction [20]. An important feature of ILs is their melting temperature and the ease of mixing them with water or organic solvents (solubility) [12]. These properties can be controlled by changing the length of the chains in cations and the type of anion [2]. For this reason, ILs are often referred to as designer solvents, and they are adapted to the processes in which they are used. These properties also depend on the structure of the ions, and with small structural changes, they can be freely changed [2]. 

The melting temperature of ILs depends also on the length and method of branching of the alkyl substituent at the quaternary nitrogen atom [12]. The first region for the methyl and ethyl substituents, the second for the alkyl of three-to-nine carbon atoms [13] and the third for above 10 carbon atoms. Melting temperatures below 0 °C are observed for liquids containing an alkyl substituent of three-to-nine carbon atoms [13]. These compounds are liquids at room temperature. Ionic liquids with low melting temperatures usually consist of a large and unsymmetrical cation (e.g., 1-alkyl-3-methylimidazolium, 1-alkylpyridinium, *N*-methyl-*N*-alkylpyrrolidinium) and one of the wide range of anion [21,22]. 

The solubility of ILs are completely different from those of other chemical compounds known to date (including traditional salts) [14]. Ionic liquids, although created by combining cations and anions, cannot be converted into molten salts [14]. The liquid state is not caused by the presence of a solvent, e.g., water [14]. Therefore, they have become an attractive subject for research in so-called green chemistry (nontoxic to the environment or living organisms) [3,23]. The most important use of Ils is to use them as green solvents, which can be used in the separation processes, synthesis, catalysis, and electrochemistry in extraction and micro-extraction processes, successfully replacing toxic and flammable classic organic solvents [18,23,24,25,26]. The above-mentioned physicochemical properties of ionic liquids can be used in many areas of our lives, as shown in Figure 1 [8,9,11]. The model of interaction of cations and anions in ionic liquids on microbial cells was also made to investigate their unique aggregation into membrane components [27]. It has been found that with long side chains of cation groups they can aggregate together to form a spatial heterogeneous region. In contrast, the leading (main) groups of cations and anions diverge evenly [27,28]. This is due to specific interactions between electrostatic interactions of charged cations and anions of the main and side groups, respectively [28]. The observed aggregation may be helpful in explaining many observed physical phenomena in ionic liquids occurring on the surfaces of the analyzed cells [28]. The bioengineering simulation studies of ionic liquid-biomembrane interactions demonstrate the spontaneous insertion of cations or anion into the lipid bilayer and can change the structural and dynamic properties of the bilayer lead to their permeability [16]. Similar processes were observed in fungal conidia of *Aspergillus nidulans* [29]. In *A. nidulans*, ILs damage the filaments and cell wall of both fungal conidia [30]. 

In other studies, they were tested in various in vitro and in vivo conditions the antimicrobial activities for three alkyl [(1R,2S,5R)-(−)-menthoxymethyl] dimethylammonium chlorides for a set of bacteria and for the wild type *C. albicans SC5314* [31]. Obtained results suggested a strong effect of the alkyl substituent chain length on the biological activity [31]. Based upon the cited studies biological activities of ionic liquids, useful in medicine, and conditioned by the following:

- Application of long alkyl substituents in a cation [11,16,32],
- Application of an alcohol molecule as a cation (antifungal activity) [33],
- Application as an anion of a drug molecule, such as acetylsalicylic acid or a cytostatic drug [34,35],
- Application of acyclic nucleoside analogs (antivirus activity) [36],
- Blending with graphene powder [37].

At present, the interest is growing in the application of ILs as agents demonstrating also antiviral, anticancer, and antifungal activity [35,36]. The application of acyclic nucleoside analogs as antiviral drugs was used for the synthesis of 3-aminoimidazo[1,2-α]pyridine in the environment of reactions 1-Butyl-3-methylimidazolium bromide [bmim][Br] [35,36]. The compound is characterized by a high antiviral activity and the reaction of obtaining takes place with a high 70%–90% efficiency at room temperature. Ionic liquids are also applied as components of anticancer drugs [35]. The presently applied boron neutron capture therapy uses l-4-boronophenylalanine, which is obtained by using the following solvents: [bmim] [BF_4_] or [PF_6_]. Chemotherapy is characterized by a selective accumulation of ^10^B isotope in rapidly dividing cancer cells [35]. Studies have shown that microscopic images of analyzed tissues stored in ILs are 10 times more pronounced than in the case of traditional solvents, e.g., formaldehyde [5,21]. This feature may be useful in the future for faster and more precise diagnosis of various inflammatory conditions or pre-motor changes. The antifungal activity of ionic liquids has not been thoroughly studied yet [38]. Based on experiments conducted with the use of menthol molecule in an ionic liquid, it has been demonstrated so far that the following mechanisms toxic for *Candida albicans* fungi takes place: Dissolution of fungus cell wall (consisting of chitosan), surfactant activity of a cation molecule, which intercalates into the phospholipid membrane, and a blockade of efflux pomp ATP-binding cassettes [16,33].

### 1.2. Divisions of ILs

**Figure 2:**
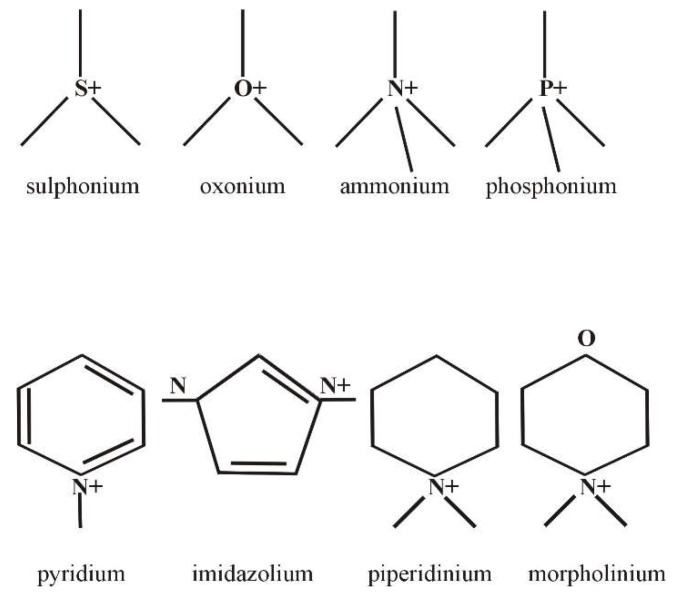
Examples of cations in ionic liquids.

The interest in ionic liquids for medicine and science has caused the group to expand rapidly with new compounds [39,40]. Therefore, it was necessary to introduce a classification dividing ionic liquids based on the type of cation and anion, and the differences in the physical properties related to their physical state [40]. The occurrence of ILs in the liquid state at relatively low temperatures is due to the presence of structures that inhibit crystallization in their molecules [39]. Among them, hydrogen bonds between the cation and anion can be distinguished, as well as significant size and strong asymmetry of the organic cation [39]. There are described ILs containing a cation in which the positive charge is located on the nitrogen, phosphorus, sulfur, or oxygen atom [40]. On this basis, ammonium (known as tetraalkylammonium), phosphonium, sulphonium, and oxonium ionic liquids were divided based on the presence of an aromatic ring in their structure, including pyridine, imidazolium, piperidinium, and morpholinium (Figure 2) [40].

**Figure 3:**
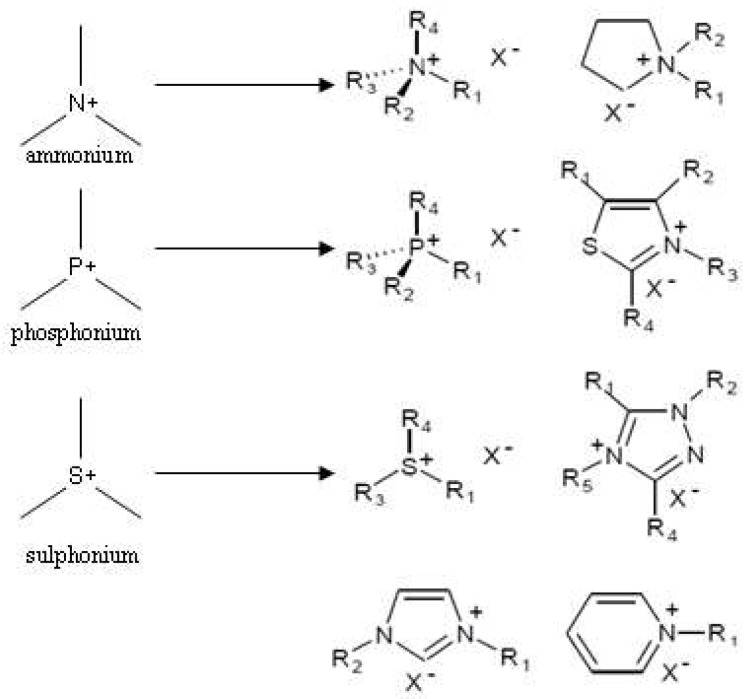
Cations and anions of ionic liquids with their structure and an exemplary type of substituent. X means: Tetrafluoroborate –BF_4_^−^, trifluoroacetate CF_3_COO^−^, chloride –Cl^−^, hexafluoroantimonate –SbF_6_^−^, bis trifluoro sulfonyl imide –(CF_3_SO_2_)_2_N^−^, trifluorosulfane acetate –CF_3_SO_3_^−^ [46,47].

In current research, ionic liquids with ammonium, imidazolium, pyridinium, and phosphonium cations are the most popular and best described, while the least-known ones remain oxonium ionic liquids, many of which are metastable [40]. In contrast, sulfonium ILs, due to the unpleasant odor of the original thiols, are not current objects of research. The essence of the general division of ILs is based on the type of atom that possesses a positive charge [18,40]. The cation may contain one or more positive atoms. Examples of multicore ammonium ionic liquids incorporate cations in which there are three positive nitrogen atoms, referred to as trigeminal tricationic ionic liquids [18]. There are a huge number of possible combinations of cations and anions (it is estimated that their number may even be 10^18^), [18] and because interest in ionic liquids is constantly growing, it is difficult to divide them including all of their aspects (Figure 3) [18]. Ammonium ILs are characterized by sp^3^ hybridization and imine sp^2^ hybridization. For ILs, we include, among others, imidazolium and pyridine with a particular type of substituent of the groups R_1_–R_6_ [41]. The R_1_–R_6_ groups can be a proton or an alkyl group or other groups, e.g., alkoxymethyl or alkylthiomethyl groups. An interesting example is imidazolium ionic liquids [41]. Due to the flat structure of the imidazole ring, they easily retain symmetry in the molecule when the R_1_ and R_2_ groups are identical. However, the difference in structure between the alkyls R_1_ and R_2_ determines the asymmetry of the cation [42]. The classification of the cation is also determined by differences in the structure. A positively charged atom may have distinct and non-interconnected alkyl substituents, which determines a large variety of conformations of such a cation and directly affects its physicochemical properties [43]. Such cations are referred to as aliphatic. The opposite of these cations are heterocyclic cations, in which the charged atom is one of the elements of a cyclic or polycyclic group with condensed rings [43]. Among heterocyclic ammonium cations, two subtypes of structures can be distinguished depending on the hybridization of the charged nitrogen atom [43]. On the basis of this criterion, heterocyclic cations can be divided into cations containing a heteroatom of sp^2^ hybridization (aromatic heterocyclic cations, e.g., imine) or of sp^3^ hybridization (non-aromatic heterocyclic cations, e.g., ammonium) [44]. Regardless of the type of element on which the positive charge is located in the cation structure, the next criterion of division is distinguished [44]. If the atom with a positive charge is chemically bound to at least one hydrogen atom, salts containing a cation of this structure are referred to as protic ionic liquids (PrILs) [44]. The presence of a hydrogen atom as the central atom of the cation leads to the formation of a network of strong supramolecular hydrogen bonds, the presence of which is a characteristic of PrILs [44]. If a positively charged atom is not connected to any hydrogen atom, i.e., it has a maximum order, then such an ionic liquid is called an aprotic ionic liquid (AIL) [23,44,45]. Initially, research on this group of compounds focused on their use as electrolytes in thermal batteries, but their use in electrochemistry is not limited to this purpose [23].

Classification of ILs may be carried out taking into account their structure, properties or application. Division due to the construction of the anion is conducted mainly due to the nature of the anion, which can be organic and inorganic [18]. In the case of organic anions, it is also possible to extract anions in which the negative charge is located on the oxygen atom of the carboxyl group (acetates, formates, citrates, and lactates) or sulfonate (methylsulphates (VI), benzenesylsulfonates, cyclamines) or on the nitrogen (azolanes, acesulfams). Inorganic anions include nitrates (V), tetrafluoroborates, hexafluorophosphates, chloroborates, or chloroaluminates, among other bisulphates (VI) [48]. 

A much more important classification of ILs is the division proposed in 2007, dividing ILs into three generations that distinguish their properties and applications, as described by Yadav et al. [26].

Biological activity may be based on both cations and anions, and it is possible to synthesize multifunctional ILs of the third generation [49]. The ions often used in pharmaceuticals are a source of both cations and ILs anions for the 3rd generation, operating as analgesic or anti-inflammatory agents, among other uses [49]. This generation also includes plant protection products, including proton triazoles with fungicidal activity and herbicidal ionic liquids (HILs), e.g., phenoxy-acid derivatives showing selective herbicidal activity against dicotyledonous plants [49]. 

The local application of ILs is an excellent alternative to organic solvents due to the effect exerted on fungal cells, bacteria, and protozoa, while not having a toxic effect on mammalian cells at the therapeutic concentration [50,51,52]. At present, the third generation of ILs has been developed, which are referred to as active pharmaceutical ingredients (APIs) [11]. Restrictions on the use of agents as standards for decontamination also result from inactivity against viruses, especially those transmitted in surgical wards [51]. Difficulties in combating viruses were associated with massive hepatitis B and C virus infection outbreaks (HBV, HCV) in the 1980′s and 1990′s of the 20th century [53]. In neonatal wards, the main problems are contact viruses, such as influenza virus, rotavirus, and norovirus [51,53]. ILs also show antiviral activity in addition to antibacterial activity [52,53]. The antiviral activity in the experimental model, based on the MS2 and p100 viruses, consists of cation activity in the ILs structure, which is responsible for the toxic effect [51,53]. Increasing the cationic side-chain length leads to increased antiviral activity of [DODMA][Cl] and [TMC_8_A][Cl], of course, until the so-called “cut-off effect” is obtained, after which no further degradation of virions is obtained [50]. At present, the accepted consensus is that the side chain length is the main indicator of the biological activity of ILs, which results from increased lipophilicity [54].

### 1.3. Synthesis of ILs

Interest in proton ILs based on the presence of an “acidic” proton in the cation contributed to the preparation of new methods to produce ionic salts [22]. ILs can be obtained in a one-step or two-step reaction. The one-step synthesis consists of the reaction of an amine with a quaternary agent, such as methyl trifluoromethylsulfonate or dialkyl sulfate [55]. The synthesis of 1-ethyl-3-methylimidazolium trifluoromethyl sulfonate is a classic example of a one-step reaction, and the reaction product is an aprotic ionic liquid [22,55]. The reaction proceeds with a high yield and is characterized by the absence of a by-product. In one-step synthesis methods, proton ILs can also be obtained by reacting a tertiary amine with an acid [22,55]. Currently, the universal method of obtaining ionic liquids is a two-stage synthesis. In the first stage, the amine reacts with a quaternary agent, which results in a quaternary ammonium chloride or bromide [26,39]. The resulting halide is often treated as a precursor of the ionic liquid. In the second stage, there is a reaction consisting of exchanging the halide with another anion in solution or in an ion exchanger, also in reaction with a Lewis or Brönsted acid [49]. An important element in the process of obtaining ILs is their purification method [49]. Due to the ionic structure, distillation cannot be treated as an effective purification method, although it is possible [26,49]. The ion exchange reaction, often called the metathesis reaction, takes place with a high yield, which is influenced by the type of solvent used and the temperature.

### 1.4. Pollution of ILs

The main contaminants of ILs are inorganic salts, resulting from the anion exchange reaction. The most popular method the cleaning of hydrophobic ILs is multiple rinsing of halides with distilled water until the disappearance of halide ions, monitored using silver nitrate [3]. This method works best with tetrafluoroborates, hexafluorophosphates and bis(trifluoromethylsulfonyl)imides. Finally, an anhydrous liquid is obtained by simple phase separation, washing with water, and finally, drying the product under a vacuum. The method of purification of hydrophilic ILs is more difficult. These include liquids containing the anions CF_3_COO^−^, CF_3_SO_3_^−^, and N(CN)_2_^−^. Purification involves dissolving a hydrophilic liquid in anhydrous acetone, acetonitrile, chloroform, or methanol [3,56]. In this case, the salt by-product does not dissolve in the anhydrous organic solvent; therefore, it is separated by filtration. After distilling the solvent, the resulting liquid is dried under reduced pressure at 60–80 °C for at least eight hours [21]. The second contaminant after inorganic salt is water. It has been assumed that in dried ILs, depending on their structure, the water content should vary between 200–400 ppm or even over 1000 ppm and several thousand ppm [57,58]. Interest in proton ILs based on the presence of an “acidic” proton in the cation contributed to the preparation new methods to produce ionic salts [57]. ILs can be obtained in a one-step or two-step reaction. The one-step synthesis consists of the reaction of an amine with a quaternary agent, such as methyl trifluoromethylsulfonate or dialkyl sulfate [57,58]. 

### 1.5. Toxicology

Quaternary ammonium halides, precursors of ILs, are known for their antimicrobial properties. They show relatively low toxicity in relation to warm-blooded organisms and have been used for years in sterilization, disinfection, bactericidal, and fungicidal preparations [22,59]. They are most often used as antiseptics, as in benzalkonium chloride and bromide, chloride didecyldimethylammonium, and hexadecylpyridinium chloride [59,60,61,62]. Quaternary ammonium chlorides and bromides have some drawbacks, including that they are bitter in taste. Exchanging the chloride anion for acesulfamate or saccharinate causes the salt to become sweet [59,60,61,62]. Acesulfamates and saccharates could therefore successfully replace chlorides wherever contact occurs in oral preparations and antibacterial mouth rinses. It has been shown that alkoxymethyl (2-hydroxyethyl) dimethylammonium acesulfamates, and alkoxymethyl (2-ethanoyloxyethyl) are water-soluble and have strong biological effects [59,60].

## 2. Antimicrobial Properties of a Quaternary Ammonium Halide

ILs, due to their properties, dissolve many different chemical compounds, and are soluble also in many solvents (as deep eutectic solvents) [63]. It has been shown that many organic reactions can be carried out in ionic liquid, with reactions that can also be carried out in chemical and separation processes simultaneously. The ionic liquid can be a good reaction medium for nucleophilic substitution, electrophilic addition, as well as electrophilic substitution in antimicrobial properties and mechanisms of action on bacterial cells [58,59]. There are four basic mechanisms of action of chemical compounds on bacterial cells: Denaturation of proteins and disruption of nucleoprotein complexes, damage to the cell membrane, oxidation of sulfidryl groups, and reactions with amino groups [39,48]. Chemicals used as active substances in disinfectants have a much wider range of activity than do antibiotics [39,48]. They are usually used in higher concentrations and are less selective and often attack multiple targets in microbial cells. An example of this is quaternary ammonium halides, responsible mainly for the disorganization of the cytoplasmic membrane of bacteria and the plasma membrane of fungi. The mechanism of action is multistage. In the first stage, the cation interacts with the negative structural proteins of the outer membrane of bacteria [48]. The cations adsorbed on the surface of the cell penetrate through the cell wall, connecting to the cytoplasmic membrane, damaging its semipermeable structure in a selective manner and then penetrating into the interior of the cell. As a result of these processes, cell outflows of potassium, sodium, phosphate and purine, pyrimidine and pentose ions occur [64]. The activity of respiratory enzymes, including succinate dehydrogenase and cytochrome oxidase, is inhibited, and oxygen consumption in the cell decreases [65]. Disruption of glycolysis and the synthesis of nucleic acids and proteins occurs, as well. Cell lysis may also occur as a result of activation of autolytic enzymes [65]. All presented changes occur when the concentration of quaternary ammonium halides is high. They are irreversible transformations and lead to cell death. Low concentrations of these compounds cause reversible disturbances of cell division, enzymatic processes, and glycolysis [65]. Effects on the processes inside the cell may vary depending on the type of quaternary ammonium halide and the type of microorganism [39,48].

The bactericidal and fungicidal activities of the compounds depend primarily on the structure of the cation, and in particular, on the length of the alkyl or alkoxymethyl substituents [56]. The antimicrobial activity starts when the alkyl chain contains eight and more carbon atoms [66,67,68].

Extending the chain with subsequent carbon atoms results in an increase in the activity between 12–18 carbon atoms, where the maximum activity is observed, and usually falling with an increase to 16 carbon atoms [66,67,68]. The change of anions for the same cations usually does not affect the biological activity. The biocidal properties of ILs allow their use as disinfectants, antibacterials, and fungicides [33].

### Interaction Quaternary Ammonium Halides with Different Pathogenic Bacterial Strains Often Occurring in Nosocomial Infections 

Bactericidal activity occurs only against the vegetative forms of both types of bacteria, among which Gram (+) bacteria are more sensitive than Gram (−) bacteria. The highest activity of ionic liquids is seen in relation to Gram (+) cocci (e.g., from the genus *Staphylococcus*, *Streptococcus*) and other Gram (+) bacteria, including *Lactobacillus* and vegetative forms of *Bacillus subtilis*. The antimicrobial activity is significantly dependent on the length and number of alkyl chains in the molecule [39,48,69,70]. The activity is the highest for compounds that contain from 10 to 16 carbon atoms in the alkyl chain or from eight to 14 carbon atoms in the alkoxymethyl group. There are no differences in the action of halogens containing quaternary nitrogen sp^3^ or sp^2^. Therefore, the activity of quaternary ammonium halides is comparable to that of pyridine or imidazolium halides in terms of the length of the alkyl substituent in 3-alkoxymethyl-1-imidazolium chlorides on the effect towards the ESKAPE group bacteria [71]. High biological activity is observed for up to 12 carbon atoms in both substituents. An increase in the number of carbon atoms above 12 causes a marked decrease in the activity and an increase in the hydrophobicity of the chloride being tested [71]. Among fungi, yeasts (including *Candida albicans*) and filamentous fungi (e.g., *Aspergillus niger*, *Chaetomium globosum*, *Myrothecium verrucaria*, *Trichoderma viridae*, *Coniophora puteana*, and *Trametes versicolor*) were susceptible to quaternary ammonium halides [71].

*Klebsiella pneumoniae* deserves special attention. Bacteria from the *Klebsiella* genus are Gram (−) enterobacteria of the family *Enterobacteriaceae*. This bacterium may be a component of the physiological bacterial flora of the digestive tract but also of the skin and mouth, especially among medical personnel [72]. In people with immunodeficiency, this species can cause severe infections, including urinary tract infections, liver, sepsis, soft tissue infections, and peritonitis. It was also described as the cause of pneumonia for the first time. *K. pneumoniae* is the most frequently isolated species of the *Klebsiella* genus in Poland (about 95% of isolates) [72]. Bacteria of this species may also be present in the environment, including in water, sewage, soil, and plants. As described in the first part of this article, the most dangerous from the medicinal point of view is *K. pneumoniae* ***N****ew **D**elhi **m**etallo-β-lactamase-1* (NDM-1) strain infection. New Delhi metallo-β-lactamase type 1 is an enzyme from the group of metal-β-lactamases that makes the bacteria resistant to the β-lactam antibiotics spectrum. The enzymes have the ability to inactivate penicillins, cephalosporins, and carbapenems. Usually, sensitivity to monobactams remains [73]. Strains exhibiting such a resistance mechanism, named KPC (*K. pneumoniae* carbapenemase), were characterized recently in Poland, Italy, and other EU countries [73]. To date, 16 different genes encoding carbapenemase have been identified in *K. pneumoniae* [74]. The object of the widest research is currently KPC-2 and KPC-3, coded by the ^bla^KPC-2 and ^bla^KPC-3 genes [75]. *K. pneumoniae* bacteria are usually resistant to penicillins because the production of β-lactamases is encoded by genes found on the plasmids present in the cell. The gene encoding the NDM-1 enzyme has been also detected on a plasmid that easily translocates to *E. coli* strains [76]*.* Genes coding the proteins for resistance to most of the available antibiotics have also been detected on the same plasmid [76]. Bacteria of this species are commonly found in the environment, in addition to as physiological intestinal flora [77]. In addition, *E. coli* bacteria demonstrates extraordinary ease in taking drug resistance genes from other species, which may lead to the assembly of other resistance genes in their cells in the near future. NDM-1 was identified in *K. pneumoniae* and *E. coli*, and was isolated in 2008 in a hospital patient in New Delhi (India) from a patient diagnosed with a urinary tract infection [77]. The reasons leading to creation of a reservoir of NDM-1 strains on the Indian subcontinent are a favorable climate, overgrowth, and the tendency to abuse antibiotics, for which purchase in India since 1 March, 2014 only requires a medical prescription [78]. Later, NDM-1 appeared in several countries such as Pakistan and Bangladesh, from where NDM-1 was imported to Great Britain, the United States, Canada, Japan, and Brazil. In 2009, it was found in a patient in Sweden who returned from a trip to New Delhi [77]. Bacteria isolated also from urine were resistant to almost all available antibiotics, with the exceptions of colistine, and tigecycline. Since August 2010, bacteria that produce the NDM-1 enzyme have been rapidly spreading in many countries on all continents, causing lethal infections. The first death occurred in Belgium in June 2010 in an ICU patient [79].

ILs may constitute the first line of defense against *K. pneumoniae* NDM-1 infections [80,81]. The first work on the impact of the organic anion showed that ILs are effective in combating bacterial biofilms in the respiratory and urinary tract [82,83]. It has been demonstrated that the cation in ILs mainly determines the biodegradability and toxicity to aquatic organisms [82,84]. ILs with short substituents of one-to-five carbon atoms that are relatively less toxic than liquids with substituents of seven and more carbon atoms [84]. This dependence also applies to biodegradability. ILs with short alkyl substituents are more biodegradable. The acesulfam didecyldimethylammonium and didecyldimethylammonium saccharin used for the studies, even at higher doses, were found to be nontoxic [61]. Ammonium ILs exhibit antielectrostatic properties and can be successfully used as both external and internal antielectrostatic agents [61]. 

## 3. The Use of Ionic Liquids in Microbiology and Medicine

The antibiotic resistance phenomenon in microbiology and medicine is associated with the use of drugs that have a bactericidal or bacteriostatic effect. Due to acquiring increasing tolerance to the drug in microorganisms, strains resistant to treatment are developed, requiring further, long-term antibiotic therapy and the simultaneous use of antibiotics from different groups [24]. Therefore, the treatment of resistant bacteria is associated with hepatic and renal toxicity, long-term hospitalization of patients and significant costs incurred by the European Union’s health care system [85]. The cost associated with hospitalization and treatment of a patient infected with antibiotic-resistant bacteria is higher than 10.000 to 30.000 USD, compared to lesser costs incurred for patients treated for infection caused by β-lactams-sensitive bacteria [86]. This emerging problem is recognized by the North American and EU disease control agencies. Moreover, ECDC reports state that nosocomial infections caused by *Staphylococcus aureus*, *Escherichia coli,* and *Pseudomonas aeruginosa* are responsible for 110.000 deaths per year in all EU countries (see Table 1) [85,87]. 

Therefore, new, rational methods of treatment are sought to ensure a reduction in development of drug resistance phenomenon. The World Health Organization states that antimicrobial resistance is a global problem due to the increasing incidence of infections with priority pathogens and persisting mortality, despite the introduction of new antibiotics [91]. We include the following bacteria in the above group of microorganisms constituting a danger for social health: *Enterococcus faecium*, *Staphylococcus aureus*, *Klebsiella pneumoniae*, *Acinetobacter baumannii*, and *Pseudomonas aeruginosa*, collectively referred to by the acronym ESKAPE [88,92,93]. Infections with bacteria belonging to the *Enterobacteriaceae* family are the main cause of morbidity and death in the USA and EU countries [88]. According to the recommendations of the Center for Disease Control and Prevention (CDC, the USA), infection with *Escherichia coli* (*E. coli*) and *Klebsiella pneumoniae* strains resistant to treatment with third generation cephalosporins are treated as a threat to public health [94].

The antibacterial activity of ILs results from the electrostatic affinity in relation to the membranes surrounding the bacterial cell wall. All prokaryotic organisms, both Gram (+) and (−), have a negatively charged surface [39]. In the case of Gram (+) bacteria, the glycolic polymer sheath based on teichonic acid is responsible, and in the case of Gram (−) rods, this sheath consists of a lipopolysaccharide endotoxin bound to a phospholipid bilayer membrane in which zwitterionic compounds are anchored [39,48]. The antibacterial activity of ILs is due to the interaction of the alkyl chain with the lipid membranes of cells, leading to the formation of ion channels, and as a result, to the disturbance of the intracellular potential and bacteria death [47,94,95].

The structure and bactericidal properties of ILs can be modified by the addition of an appropriate antibiotic molecule, e.g., ampicillin, to the anion part of ILs [39,48]. Application of ILs eliminates the issue of drug resistance [95]. However, most of the molecules that interact coulombically are initially susceptible to their activity, over the time bacteria may change membrane charge or phospholipid density. The resulting antibiotic-ILs combination is characterized by a much lower minimum inhibitory concentration MIC and *minimum bactericidal concentration* MBC compared to the antibiotic itself [51]. The combination of ILs with the drug molecule, apart from the resulting antimicrobial synergistic effect, has an impact on the drug pharmacokinetics, including parenteral absorption and tissue distribution [49,96]. The use of ILs does not induce drug resistance, which is why the possibility of effective treatment of life-threatening infections and nonhealing wounds is postulated [50,96]. The biological activity of ILs results from the modification of a cationic chain fragment and connection with the ring structure of β-lactam antibiotics. The elongation of alkyl chains in *N*-alkylimidazolium and *N*-alkylpyridinium allowed achievement of a biological effect through the alkyl chain of the ionic liquid and DNA intercalation [97]. Adducts of bacterial DNA with ionic liquids, arisen in this way, lead to inhibition of transcription, translation, and cell division processes, resulting in bacterial death [98]. Combination of ILs with the structure of commonly used antibiotics is the basic direction of development for new therapeutic substances [11,51,94,95]. The synthesized antibiotic-IL complexes are characterized by an increased spectrum of antibacterial activity, as well as better absorption and tissue penetration, which until now has not been achieved (parenteral administration of the liposomal form of amphotericin B leads to penetration through the blood-brain barrier) and much less toxicity due to the therapeutic efficacy of smaller doses of antibiotics [89]. The minimum inhibitory concentration (MIC) of the 2,3-dimethylimidaziolium molecule is reduced by more than half, from 23 mmol/L to 7.9 mmol/L, thus obtaining a value that is also the minimum bactericidal concentration (MBC). The complex compounding of ILs with precious metal ions such as silver and gold is also worth mentioning [99]. The obtained metal-IL complexes are characterized by significant antibacterial activity and a long duration of action of the drug [80]. Therefore, IL complexes with silver and gold ions are used for local application in wounds and infected surgical sites and in endodontic treatment. The described complexes are characterized by particular activity against vancomycin-resistant [VRE] *Enterococcus* strains, which are VRE alert strains [98].

*Staphylococcus aureus* is currently regarded as the most dangerous bacterium in social health. A staphylococcal carrier state is found in about 30% of hospital staff, and patients are colonized by touch [100]. *Staphylococcus* causes chronic osteoarthritis, infective endocarditis, and bacteremia and colonizes artificial materials implanted in the human body, such as hip joint prostheses, artificial heart valves and vascular stent grafts [100]. Despite the introduction of new antibiotics for treatment, including vancomycin and meropenem, the mortality rate from staphylococcal infections has not changed over the last five years [90]. The main mechanism of staphylococcal antibiotic resistance does not consist of the production of enzymes called β-lactamases, which decompose antibiotics, as in the case of *Enterobacteriaceae*, but of the synthesis of a new protein called PBP (Penicillin Binding Protein) and efflux pumps that route the antibiotic outside of the bacterial cell [90]. The described mechanisms lead to antibiotic resistance to penicillins, cephalosporins, monobactams, and carbapenems, and there is an additional phenomenon of cross-resistance to macrolides and fluoroquinolones. ILs used in the treatment of general and local infections are not susceptible to either a antibiotic-resistant mechanism, which was proven in the studies on the SA1199 A and B staphylococcal strains (see Table 2) [86,91,92,93].

ILs exhibiting antistaphylococcal activity are divided into two groups: Phosphonium ionic liquids and nitrogen ionic liquids, which include alkylammonium and/or imidazolium salts [97,98,101]. 

So far, the most effective agents for staphylococci have been the recently synthesized phosphonium ionic liquids (PILs) obtained from triphenylamine (TPA) [94,95]. Phosphonate compounds are characterized by lower MIC and MBC values, compared to ILs based on nitrogen compounds, which are less toxic to the human body and have a different mechanism of antibacterial activity [101]. The PILs complexed with TPA derivatives spontaneously form layered polymeric multicomposites that have the ability to penetrate the outer membrane and act within the bacterial cell [101]. This is a clinically significant feature because, due to their easy penetration, PILs-TPA compounds are characterized by MIC values that are unachievable even for the most effective antibiotics (0.25 μg mL^−1^ vs. 2–3 μg mL^−1^ in blood serum for vancomycin), [102,104]. Mono, di, and tri-formylation of TPA is obtained in the Vilsmeier-Hack reaction. The key stage of Williamson etherification is carried out using NaH in a solution of ethanol and chloroform. The sodium salt, thus obtained, comes into reaction with Br-(CH_2_)_n_-OTBDMS [102,104]. The group blocking the compound is then removed using tetra-n-butylammonium fluoride (TBAF) to give the appropriate alkyl chain length. The process of obtaining the alkyl chain in the TPA molecule is extremely important, as the MIC decreases for all Gram (+) cocci as the chain length increases. The MIC of TPA 22 for *S. aureus* is eight mg/L and 16 mg/L for *E. faecium*, while the MIC for TPA with a chain length of 24–27 is two mg/L for *S. aureus* and ranges from two up to eight mg/L for Enterococcus [66,80,99]. The presence of a counter ion in the molecule does not affect the antimicrobial activity. The bactericidal activity of ILs against cocci results from the rapid penetration of ILs through the glycopolymer sheath, which in Gram (+) bacteria, consists mainly of teichoic acid. ILs based on phosphonate with alkyl chains have bactericidal effects against *A. baumannii* as well as other Gram (−) bacteria [74]. This effect is, however, much weaker than that for cocci due to the presence of the lipophilic outer membrane on the outside of the cell wall of Gram (−) bacteria [74]. The MIC of TPA for *A. baumannii* is from 16 to 64 mg/L, which is four times weaker than the effect of ILs obtained against rods. This is due to the stronger bi-layer intercalation of the outer membrane, followed by the creation of a cationic channel leading to a change in the transmembrane potential, and eventually, cell death [74].

*K. pneumoniae* New Delhi has been a widespread pathogen in Asia since its discovery in 2008 in India [105]. A troubling problem is infections with pathogens such as the *Klebsiella pneumoniae* New Delhi strain, which produces metallo-β-lactamase-1 (NDM), and until now, was considered exotic in Europe [106]. *K. pneumoniae* NDM causes pneumonia, sepsis and soft tissue inflammation in humans and in transplant recipients due to hospitalization and immunosuppressive treatment [107]. The New Delhi *K. pneumoniae* strain is resistant to treatment with meropenem and other carbapenems due to the extended spectrum of β-lactamase produced by this strain, containing a zinc ion in the active centers [107]. The phenomenon of drug resistance to carbapenems and β-lactams is related to gene cassettes carried on class 1 integrons [108]. The NDM strain encoding the ^bla^NDM-1 gene is extremely easily transmitted by transfection genetic elements, as well as in the air; hence, it is responsible for rapid bacteria propagation [109]. The current drug regimen presupposes the use of colistin, aminoglycosides and trimethoprim together with sulfamethoxazole. Colistin is characterized by high nephrotoxicity, and the use of aminoglycosides is associated with an irreversible cytotoxic effect, while trimethoprim is characterized by a dose-dependent effect [108,109]. Hence, the drug concentrations used in humans have only bacteriostatic action against *K. pneumoniae* NDM. ILs have a bactericidal effect against the *K. pneumoniae* NDM strain [107]. The most effective method against this strain turned out to be the use of biopolymer forms of ILs. Based on nitrogen-compound ILs, which are room temperature ionic liquids (RTIL), with a melting point of 25 °C-including 1-ethyl-3-methylimidazolium chloride [C_2_mim][Cl] and 1-octyl-3-methyl imidazolium chloride [C_8_mim][Cl]), are the most effective against NDM-1 [70]. In addition, these compounds are assigned to the so-called “green solvents” group due to their low volatility, nonflammability, chemical structure stability at high temperatures, and low toxicity to eukaryotic cells [38,70]. ILs interactions in the course of the stationary phases of reverse-phase chromatography is complex due to participation of both the cation and anion [38,70]. Moreover, a nanocomposite filler, laponite, allowed a stronger bactericidal effect against *K. pneumoniae* NDM, which was observed according to the bacterial inhibition zone (BIZ) on agar plates [51]. The growth inhibition zone (BIZ) was 7 mm for [C_2_mim][Cl], while for [C_8_mim][Cl] in combination with the nanocomposite BIZ, the zone was nine mm [38]. The biological effects obtained by adding hydrogels to the tested imidazoline compounds enable the topical use of the compounds in medicine as dressings for wounds infected with *K. pneumoniae* NDM. In addition, the matrix may form a scaffolding for the growth of granulation tissue and promote repair processes in cases of tissue loss [38,70]. 

## 4. Polymorphism of Ionic Liquids as New Solvents in the Synthesis of Pharmacologically Active Compounds

ILs are often referred to as designer solvents, due to the possibility of modification of their polymorphic internal structure (specific cation or anion) and unique physicochemical properties. Polymorphism is the ability of a substance to occur in two or more crystalline forms that are characterized by a different arrangement or conformation of molecules in the crystal lattice. It is estimated that more than half of drugs have polymorphism. It is believed that 70% of barbiturates, 60% of sulfonamides, and 23% of steroids may exist in various polymorphic forms [110]. The existence of different polymorphic forms can have a significant effect on the drug’s effectiveness, because each form can have different physicochemical properties. For example, one of the existing polymorphisms may be more bioavailable, more stable (e.g., longer shelf life), or easier to use in a formulation than another polymorphic form. The use of polymorphic forms of ionic liquids has become an alternative solution in the synthesis of non-steroidal anti-inflammatory drugs (NSAIDs) [111]. Prawadolin (*pravadoline*), a drug classified into the NSAID group, is obtained by ionic liquid synthesis, based on a nucleophilic reaction substitution and Friedel-Crafts reaction [112].

Examples commonly used analgesic and anti-inflammatory drugs is known under the trade name Ibuprom^®^ (ibuprofen), which is commercially available mainly in the form of a racemic mixture. Literature data indicate that the enantiomer in vitro (S)—ibuprofen has about 150 times more anti-inflammatory activity than its (R) enantiomer—therefore, many biosynthetics, among others, are currently developed with the use of ILs to obtain higher enantioselectivity than with the application of conventional solvents [111].

The exact polymorphic form of the compound also affects its physical properties, such as dissolution rate, bioavailability, physical properties of the crystal, and mechanical strength [22]. Delivery of the exact dose of the compound to the body often depends on which of the several possible polymorphic forms is present in the formulation. The discrepancies of properties between different polymorphic forms usually means that one crystalline form is desired more than others [22]. 

However, obtaining a specific form may be difficult, and the search for salts with a specific crystal structure (usually to control the dissolution rate and solubility) may require many experiments. In this way, for each drug, the slightest change in the crystallization process, e.g., the crystallization solvent used, can lead to the formation of a polymorphic form which must be fully re-examined and characterized in terms of physicochemical properties [60]. The unintended production of an undesired polymorphic form may lead to a polymorphic form, which is a less-effective or even toxic form of the drug that will not be authorized without full clinical trials. Thus, the occurrence and control of polymorphism may be one of the most important challenges for obtaining a product of constant quality [17,113]. Using an ionic liquid instead of a conventional solvent, harmful to the water environment, gives new possibilities of obtaining therapeutic compounds, and also enables the significant elimination of toxic pollution [17,113]. Solvents are currently used in the synthesis of drugs exclusively on a laboratory scale, however, scientific research aimed at introducing this new one class of compounds, on an industrial scale, are carried out intensively.

## 5. Summary and Future Outlook

In contemporary society the consciousness and knowledge concerning the effects of human activities on the natural environment is growing. In this respect, the interest in green chemistry is increasing, meaning by the “green chemistry” substances which are considered to be beneficial for the natural environment. Ionic liquids based on imidazole compounds can be recognized as green solvents. This is important since the water environment is the key space for interactions of drugs of general application or local application. The combination of cation and anion allows the synthesis of ILs with specific pharmacodynamic properties directed against bacteria, viruses, and fungi. At present, there is a possibility of combining β-lactam antibiotics with dimethylimidazolium for a synergistic antimicrobial effect. 

The toxicity of ILs (antimicrobial activity) in analyzed Gram (−) and Gram (+) bacterial strains and fungi depends on the length of alkyl chain and type of cation. ILs containing alkyl chains with eight to 18 carbon atoms affect the bacterial membranes’ components and fungal cell walls and change their surface charge. Eventually they activate specific pathways of gene expression, resulting in metabolic disorders and cell death. The observed effect is particularly evident in the example of described quaternary alkylammonium salts. ILs are effective in removing bacterial biofilms, though the discovery of mechanisms of their specific action requires further studies. Our article is of special significance for researchers seeking the alternative, new drugs, other than antibiotics.

## Figures and Tables

**Table 1:** Morbidity, number of hospitalizations and mortality due to selected bacterial infections with priority pathogens causing blood stream infections in people in the European Union countries in 2012–2017.

Pathogen	Strain Characteristics	Number Of Detected Cases	Confirmed Cases Of ICU* Acquired Infections	Percentage Of Selected Strains Detected Is Surgical Site Infections	Drug Resistance	Comorbidity Index	Mortality	Reference
*Acinetobacter baumanii*	Nosocomial	712	541	4.1%	3rd eneration cephalosporins	2.8	18%	[85,87]
	MDR^+^					3.9	26%	[88]
*Klebsiella pneumoaniae*	New Delhi ^bla^NDM-1	1367	561	4.7%	3rd generation Cephalosporins, Meropenem, Vancomycin	6.4	72%	BIOCONTAM Unit
*Pseudomonas aeruginosa*	PAPI^+^	2 269	516	7.1%	Aminoglycosides, Gyrase inhibitors, Penicillin with β-lactamase inhibitor	4.8	38%	[75,80,89] EARS-Net
*Staphylococcus aureus*	MRSA	1996	631	38.1%	β-lactams, Lincosamides, Fluoroquinolones	4.5	30%	[90]
*Enterococcus species*	VRE	492	340	20.4%	Vancomycin, Teicoplanin	2.7	43.1%	[85] ECDC
other *Enterobacteriaceae*	ESBL^+^	1367	479	3.3%	β-lactams, 3rd generation Cephalosporins, Trimethoprim/Sulfomethaxazole	1.8	18.2%	BIOHAZ team, EARS-Net

Antibiotic resistance was developed on the basis of data from hospital intensive care units and swabs taken from nonhealing wounds from surgical sites. Data were compiled on the basis of surveillance reports, annual epidemiological reports, antimicrobial resistance, and healthcare-associated infections. ECDC, EARS-Net, EFSA, BIOHAZ team and BIOCONTAM Unit: Antimicrobial resistance annual report (Stockholm, April 2015; doi: 10, 2900/6928) [75,80,85,87,89,90].

**Table 2:** Listing of bacterial priority strains including *S. aureus*, *Enterococcus species*, *K. pneumoniae*, *Enterobacteriaceae* and *P. aeruginosa*, antibiotic resistance mechanisms and ionic liquids showing the best effect in the treatment of infections.

Bacterial Strain	Strain Specification	Antibiotic Resistance Chracteristics	Ionic Liquid	Principal Mechanism of Action	MIC [Mmol L^−1^]	MBC [Mmol L^−1^]	References
*Staphylococcus aureus*	HA-MRSA	MGEs^+^	[C_8_mim][Cl]	Collapse of liposomes, localized perforations in dioleoylphosphatidylcholine bilayers	116	170	[51,86,89,91,92,93,94,95,97,98,101]
	MZ100	SCC*mec^+IV-V^*	[C_4_C_1_Im]	Perforation of dipalmitoylophosphatidylcholine with phospholipid bilayers [Tf_2_N], isotopic substitution of hydrogen	188	250	
		*tsst-1*					
	BORSA	*pvl* ^+^	[Chol][Cl]		125	188	
	USA300	*icaD* ^+^	Di-But C_6_	Leakage of cellular liposomes, alternation of apolar regions by protic ionic liquids	1000		
	CA-MRSA	*hl* ^α-γ+^	Di-Hex C_6_		8	4	
	ATCC25923	NorA efflux pump	BTFLA		23	43	
	MDRSA						
			1,3-dialkiloimidazolinum	Docking complex formation with tubulin FtsZ PC190723, Increased affinity to the membrane of cancerous cells—interaction with phosphatydilserine	2.12	6.14	
			[C_1_C_1_^4^ pi][BF_4_ ]_2_		5.6	19.3	
			[Phpi][BF_4_]		1.2	4.8	
			[(C_2_)_2_(C_1_)_2_(C_1_)_2_ ^3^gu][C_2_OSO_3_]		>100	342	
	ATCC6538		C_16_M_1_Im][Br]		23	NA	
			[C_16_M_1_Im][Amp]*		7.9	7.9 **	
*Enterococcus species*	COM12-15 E1071 E4452-E4453 AUS0004	VRE	[C_12_Py]	De-stabilising effect on lipid structure	8.1	8.1 **	[38,66,69,80,99]
			[C_18_Py]		8.5	8.5 **	
			[EMIm^+^Tf_2_N^-^]	Mismatch between ionic liquid cations and lipids in the layer	16.8	34.6	
			[C_12_Im]	Long-tail cation mediated cytotoxicity, electrostatic signature interacting with peptidoglycan	7.1	7.1 **	
			[C_18_Im]		8.1	8.1 **	
			[BMIm^+^Cl^-^]		7.5	7.5 **	
			Ag^+^C_3_H_5_N_2_-*p*		5.7 × 10^−10^	5.7 × 10^−10^ **	
*Klebsiella pneumoniae*	ATCC4352	Metallo-β-lactamse-1	[C_2_mim][Cl]	Inhibition of acetylcholineesetrase	178	263	[51,96]
			[CBP]	Interaction with phosphatidic acid, apoptosis triggering	13	NA	
			[CPB][AMP] *	Antibiotic donor	4.7	9 **	
			[C_16_M_2_Im][Br]	Interaction with cytoskeleton protein subunits	15	NA	
			[C_16_M_2_Im][AMP] *	Antibiotic donor	7.8	7.8 **	
*Enterobacteriaceae genus*	W3110	OXA-48 carbapenemase	[Chol]^+^ Thre	Interaction with biomembranes surrounding cellular organelles	31.3	62.5	[94,95,102]
			[Chol]^+^ Pro		46.9	62	
		β-lactamase encoding genes K_1-2_ capsular serotypes	Di-But C_10_	Decreased flexural rigidity and reduced interfacial tension between the bilayer and ionic liquid, lysis of bacterial outer membrane	40	40 **	
			Di-Hex C_10_		8	8 **	
			PTLFS		91	470	
			[(C_2_)_2_ ^2^(C_1_)_2_(C_1_)_2_ ^3^gu][C_2_OSO_3_]		12.5	39.5	
			[C_2_pi][BF_4_]		2.38	11.8	
			[C_2_C_1_C_1_^4^pi][I]		>50	185	
*Pseudomonas aeruginosa*	PA14	OM-proteins	Di-Hex C_10_	Coagulation of cytoplasm	9	20	[50,103]
	ATCC 27853	MDEP	1,3 dialkiloimidazolinum	Asymetric absorbtion of ionic liquid cation by leaflets of phospholipid bilayer	18.4	45.8	
			BMP-NTf_2_		24	65.6	
			HMIM-Cl		12.4	61.5	
			[P(C_14_H_29_)(C_6_H_13_)_3_]^+^		8.4	20	
			(ZnCl_2_)_2_ - BZBN		12.3	31	

The mechanism of action of ILs and the minimum inhibitory concentration, and the minimum bactericidal concentration are given. * ILs, which belong to APIs, were identified. ** The inhibitory concentration is equivalent to the bactericidal concentration [38,50,51,66,69,80,86,89,91,92,93,94,95,96,97,98,99,100,101,103].

## Abbreviations

*A. baumannii*	*Acinetobacter baumannii*
Ag^+^C_3_H_5_N_2_ – *np*	imidazolium-based silver nanoparticles
AIL	aprotic ionic liquid
APIs	active pharmacological ingredients
BF_4_	tetrafluoroborate
*A. baumannii*	Acinetobacter baumannii
Ag+C3H5N2 - np	imidazolium-based silver nanoparticles
AIL	aprotic ionic liquid
APIs	active pharmacological ingredients
BF4	tetrafluoroborate
BIOCONTAM	EFSA unit on biological hazards and contaminants
BIOHAZ	the panel on biological hazards EFSA
BIZ	growth inhibitory zone
*^blaNDM-1^*	plasmid containing the metallo-β-lactamase type 1 gene sequence
BMP-NTf2	1-butyl-1-methylpyrrolidinumbistriflimide
BORSA	mecA-positive oxacillin resistant Staphylococcus aureus
BTFLA	bis trifluoromethylsulfonyl amide [(CF3SO2)2N]^−^
CA-MRSA	community-associated methicillin-resistant Staphylococcus aureus
Di-But C10	phosphonium, 1,1′-(1,10-decanediyl)bis[1,1,1-tributyl]
Di-But C6	phosphonium, 1,1′-(1,6-hexanediyl)bis[1,1,1-tributyl]
Di-Hex C10	phosphonium, 1,1′-(1,10-decanediyl)bis[1,1,1-trihexyl]
Di-Hex C6	phosphonium, 1,1′-(1,6-hexanediyl)bis[1,1,1-trihexyl]
*E. coli*	Escherichia coli
*E. faecium*	Enterococcus faecium
EARS-Net	European Antimicrobial Resistance Surveillance Network
ECDC	European Centre for Disease Prevention and Control
EFSA	European Food Safety Authority
ESBL+	extended spectrum β-lactamases
ESKAPE	acronym from Enterococcus faecium, Staphylococcus aureus, Klebsiella pneumoniae, Acinetobacter baumannii and Pseudomonas aeruginosa infections
EU	European Union
FtsZ	prokaryotic homologue to the eukaryotic protein tubulin
HA-MRSA	hospital-associated methicillin-resistant Staphylococcus aureus
HILs	herbicidal ionic liquids
*hlα-γ+*	α-γ haemolysins encoding genes
HMIM-Cl	1-hexyl-3-methylimidazolium chloride
*icaD+-*	gene relevant to bio-film formation
ICU	intensive care unit
ILs	ionic liquids
*K. pneumoniae*	Klebsiella pneumoniae
KPC	Klebsiella pneumoniae carbapenemase
MBC	minimal bactericidal concentration
MDEP	mucoid exopolysaccharide strain of Pseudomonas aeruginosa
MDR+	multi-drug-resistant Acinetobacter baumannii
MDRSA	multi-drug-resistant and methicillin-resistant Staphylococcus aureus
MGEs+	mobile genetic elements encoding methicillin resistance
MIC	minimal inhibitory concentration
MS 2 virus	Enterobacteriophage type 2
*NorA*	Norfloxacin efflux pump gene
NSAIDs	non-steroidal anti-inflammatory drugs
OTBDMS	O-tert-butyldimethylsilyl
p100 virus	Listeria phage 100
PAPI+	Pseudomonas aeruginosa pathogenicity islands of strain 14
PBP	penicillin binding protein
PF6	Hexafluorophosphate
PILs	phosphonium ionic liquids
ppm	parts per million
PrILs	protic ionic liquids
PTLFS	p-toluenesulfonate [4MePhSO3]-
*pvl+*	Panton-Valentine gene encoding leucocidin
RTIL	room-temperature ionic liquids
*S. aureus*	Staphylococcus aureus
SCCmec+IV-V	staphylococcal mec chromosome cassette type IV and V
TBAF	tetra-n-butylammonium fluoride
TGA	thermal gravimetric analysis
TPA	triphenylamine
*tsst-1*	toxic shock syndrome toxin 1 gene
USA	United States of America
USD	United States dollar
VRE	vancomycin-resistant enterococci
[(C2)2(C1)2(C1)23gu][C2OSO3]	2-ethyl-1,1,3,3-tetramethylguanidinium ethyl sulfate
[(C2)22(C1)2(C1)23gu][C2OSO3]	2,2-diethyl-1,1,3,3-tetramethylguanidinium ethyl sulfate
[BMIm+Cl-]	butylmethylimmidazolium chloride
[C12Im]	C12 alkyl imidazolium
[C12Py]	C12 alkyl pyridinium
[C16M1Im][Br]	1-hexadecyl-3-methylimidazolium bromide
[C16M2Im][Br]	1-hexadecyl-2,3-dimethylimidazolium bromide
[C18Im]	C18 alkyl imidazolium
[C18Py]	C18 alkyl pyridinium
[C1C14pi][BF4]2	1,4-dimethylpiperazinum bis(tetrafluoroborate)
[C2C1C14pi][I]	1-ethyl-1,4-dimethylpiperazinium iodide
[C2mim][Cl]	1-ethyl-3-methyl imidazolium chloride
[C2pi][BF4]	1-ethylpiperazinium tetrafluoroborate
[C8mim][Cl]	1-octyl-3-methyl imidazolinum chloride
[Chol][Cl]	choline chloride
[CPB]	cetylpyridinum bromide
[DODMA][Cl]	1,2-dioleyloxy-3-dimethylaminopropane chloride
[EMIm+Tf2N-]	ethylmethylimmidazolium bis(trifluoromethylsulfonyl)imide
[P(C14H29)(C6H13)3]+	tetralkylophosphonium oleate
[Phpi][BF4]	1-phenylpiperazinum tetrafluoroborate
[TMC8A][Cl]	trimethyloctylammonium
(ZnCl2)2	BZBN benzethonium chloride
*	ampicillin-based ILs
**	The MIC value was equal to the MBC of ampicillin-based ILs.
*[C16M1Im][Amp]	1-hexadecyl-3-methylimidazolium ampicillin
*[C16M2Im][AMP]	1-hexadecyl-2,3-dimethylimidazolium ampicillin
*[CPB][AMP]	ampicillin-based ILs cetylpyridinum
